# Kinship as a frequency dependent strategy

**DOI:** 10.1098/rsos.150632

**Published:** 2016-02-17

**Authors:** Ting Ji, Xiu-Deng Zheng, Qiao-Qiao He, Jia-Jia Wu, Ruth Mace, Yi Tao

**Affiliations:** 1 Key Laboratory of Animal Ecology and Conservation Biology, Centre for Computational and Evolutionary Biology, Institute of Zoology, Chinese Academy of Sciences, Beijing 100101, People’s Republic of China; 2 Department of Anthropology, University College London, 14 Taviton Street, London WC1H 0BW, UK

**Keywords:** kinship, post-marital residence, frequency-dependent selection

## Abstract

Humans divide themselves up into separate cultures, which is a unique and ubiquitous characteristic of our species. Kinship norms are one of the defining features of such societies. Here we show how norms of marital residence can evolve as a frequency-dependent strategy, using real-world cases from southwestern China and an evolutionary game model. The process of kinship change has occurred in the past and is also occurring now in southwestern China. Our data and models show how transitions between residence types can occur both as response to changing costs and benefits of co-residence with kin, and also due to the initial frequency of the strategies adopted by others in the population: patrilocal societies can become matrilocal, and neolocal societies can become duolocal. This illustrates how frequency-dependent selection plays a role both in the maintenance of group-level cultural diversity and in cultural extinction.

## 1. Introduction

A key feature characteristic of humans is that we divide ourselves up into cultural groups, defined by their cultural norms. Cultural norms can arise as adaptations to a particular environment, but are also, almost by definition, influenced by the behaviour of those around you. Anthropologists have always been interested in behaviour that varies at the level of the cultural group; indeed the subject defines itself in terms of understanding cultural difference. While, after years of frustrating debate, social anthropologists have largely lost interest in defining culture, evolutionary anthropologists have become more interested in how selection could be acting on cultural differences. Cultural phylogenetics was one emerging field that increased interest in which traits could be considered as properties of the group and how or whether cultural evolution was generating strategies as adaptations to different ecological conditions [1,2]. The idea that cultural traits might be group selected has emerged as a hotly debated topic in recent years, focusing interest on whether group difference can be maintained in such a way that cultural group selection could occur [3,4]. Punishment and conformism are two possible mechanisms which could maintain cultural differences between communities even when there is some migration between them [5]; although which evolutionary processes could generate punishment or conformity are debated. Here we focus on another evolutionary process that can maintain differences between groups, which is frequency-dependent selection.

Humans are unusual in exhibiting a wide range of sex-specific dispersal strategies, resulting in the emergence of different types of residence, often evident even in the same world region. These kinship norms are generally group-level traits, which have long been a focus of anthropological research.

Human kinship and residence systems can be partly defined by whether males or females disperse or stay in their natal homes, which results in four main outcomes.

(i) *Patrilocality* Female dispersal with males staying in their natal home or home area is the most common human pattern, resulting in patrilocal residence and kinship systems that often include patrilineal descent of property, wealth and titles. Patrilineal systems are often associated with resources that can be monopolized by males to attract females, and are often also associated with polygynous marriage [6].
(ii) *Neolocality* If male dispersal occurs and is associated with female dispersal, it results in neolocal post-marital residence for the married couple.
(iii) *Matrilocality* In a minority of cases, the female stays in her natal home and the male disperses to join her, resulting in matrilocal residence. Matrilocal residence is associated with matrilineal descent which, although fairly rare, is observed on all continents [7] and accounts for about 17% of those societies listed in Murdock’s Ethnographic Atlas [8]. Matriliny is generally associated with a lack of monopolizable resources, weak marriage bonds and possibly with long male absences [9].
(iv) *Duolocality* The final combination is that neither sex disperses. It is very rare for both sexes to stay in their natal homes throughout life, as this necessitates mating outside the home, but such duolocal systems where couples practice ‘walking marriage’ do exist in some rare cases [7]. The Mosuo of southwestern China are one of these groups [10,11].

The sex that does not disperse tends to experience the most competition within the family, usually among the same sex; indeed avoiding reproductive conflict between kin may be one of the reasons kinship systems have evolved [12]. This competition influences both patterns of reproduction and labour [13–15]. Dispersal patterns can cause relatedness to the group to vary with age and sex, and some argue the female relatedness to the group generated by dispersal in either patrilocal or duolocal groups has led to the evolution of menopause in humans and killer whales [16,17].

Kinship systems are not necessarily fixed over time and can be dynamic. For example, in the Bantu in sub-Saharan Africa there appear to have been frequent changes between matrilocal and patrilocal residence over the last few thousand years, in part, in response to changes in subsistence [18,19]. Phiri describes how contact between the matrilineal Chewa and the patrilineal Ngoni is thought to have changed some Chewa communities from matriliny to patriliny and some Ngoni communities in the other direction from patriliny to matriliny in certain places [20]. However, the evolutionary mechanism behind this kind of kinship transition is not fully understood.

Here we report two recent cases of kinship and residence norms changes in southwestern China, and use an evolutionary game model to illustrate how the frequency-dependent selection acts on the transition. As in Richard Dawkins’ discussion of the battle of the sexes [21], an optimal mating strategy for one sex depends in part on what others are doing, as well as on the costs and benefits associated with each possible outcome. In our model, we assume males and females each decide whether to disperse or stay in their natal home at marriage. The marital residence patterns that will emerge depend on the frequency of their own and the other sex’s dispersal decisions. We examine the evolutionary dynamics of different post-marital residence norms, by modelling the conditions under which different frequencies of male and female dispersal evolve. Our model shows how norms of dispersal can generate different patterns of post-marital residence, as a function both of the costs and benefits to an individual and of the frequency of the strategies adopted by others in the population.

## 2. Methods

### 2.1 Historical records of kinship in Mosuo and Pumi

Historical population and kinship data from Mosuo people and Pumi in Sichuan and Yunnan Provinces, southwestern China are obtained from the published data, which were collected through the survey of ethnic minorities in China between 1950s and 1970s.

Nine matrilineal Pumi villages and 23 patrilineal Pumi villages were found from historical records [22–25]. Geographical coordinates of all Pumi villages were obtained in Google Maps by matching the name of the villages. The list of Pumi villages is shown in the electronic supplementary material, table S1.

Data from 25 Mosuo populations who live in the same area with Pumi were also collected [22–36]. Among them two are from Yanyuan, one is from Muli, Sichuan, 13 populations are from Ninglang and one population from Lijiang, Yunnan. Eight localities in Lanping County are where Pumi reside but a population of Mosuo were also included. Geographical coordinates of each location were recorded by matching the name of the location on Google Maps. The list of Mosuo villages is shown in the electronic supplementary material, table S2. The contour map based on these 25 Mosuo populations was produced in RStudio (v. 0.98.1102) with the package *spatstat*, *maptools*, *sp*, *raster*, *rgeos*, *rworldmap*, *rworldxtra* and *mapdata*. The locations of matrilineal Pumi villages and patrilineal Pumi villages were then plotted on the contour map.

### 2.2 Demographic data in Mosuo in Lugu Lake Town

In 2007 and 2012, we conducted two demographic censuses in five villages in Lugu Lake Town on the shores of Lugu Lake in the Tibetan borderlands of Sichuan Province, China. Lugu Lake Town is approximately 283 km^2^, and the total population is about 10 000. Most of the inhabitants are Mosuo, and others are Yi, Han, Pumi and a few Tibetan people. For each household, one adult representative was interviewed about the demographic details of all male and female family members as well as socio-economic information (which included name, ethnic group, gender, year of birth, animal sign, education, parents’ name, marriage status, spouses’ name, children’s name, children’s year of birth, children’s gender and place of residence, GPS location, land size, number of livestock and number of hotels and businesses).

### 2.3 An evolutionary game

A natural question is what drives the evolution of post-marital residences. We here use a two-phenotype asymmetrically evolutionary game model to illustrate why the time evolution of post-marital residence could be frequency dependent. Assuming that both female and male have two possible strategies for their post-marital residence: either stay in their natal household (denoted by S) or disperse to a different household (denoted by D). Female strategies are denoted by FS and FD and male strategies are denoted by MS and MD. Based on these assumptions, four possible types of post-marital residence are (FS, MS), (FS, MD), (FD, MS) and (FD, MD), where (FS, MS) represents duolocality, (FS, MD) matrilocality, (FD, MS) patrilocality and (FD, MD) neolocality. The details of the model are presented in the electronic supplementary material.

Through dynamics analysis of the bi-matrix game (i.e. asymmetric game) [37,38], we investigate the time evolution of strategy S for both females and males given different costs and benefits associated with each post-marital residence. Model analysis and models figures were performed in Matlab.

### 2.4 Statistical analysis

We use logistic regression to predict covariates of the current residence type of married Mosuo people living in Lugu Lake Town in 2012. All statistics and figures were generated in RStudio (v. 0.98.1102) with the package *lme* and *ggplot2*.

## 3. Results

### 3.1 Transitions of kinship and residence norms in southwestern China

#### 3.1.1 Pumi minority residence within Mosuo majority areas generated matrilineal, duolocal Pumi villages

In southwestern China, the process of kinship change has occurred in the past and is also occurring now. The Mosuo (also known as the Na) inhabit strips of farmland near and around the shores of Lugu Lake in the border between Sichuan and Yunnan Provinces in southwestern China. Diets used to be supplemented by fishing and hunting, but wildlife resources are now depleted. The group is related to Pumi, Tibetans and Naxi, and speaks a Tibeto-Burman language. Mosuo families live in larger matrilineal households of three generations of brothers and sisters and the matrilineal offspring. The wives and children of the males reside elsewhere with their matrilineal kin. The marriage system is often described as ‘walking’ or ‘visiting marriage’. That is, both women and men are still living in their natal households after marriage, men visit their wives at night and return in the morning. Thus, there are usually several co-resident breeding women in Mosuo households; they are breeding communally in the sense that they cooperate with childcare, domestic and farm labour, and share all the household resources [11].

Duolocal residence is the norm in the matrilineal Mosuo. During field research in the area, we encountered a group of Pumi villages that have also adopted this rare form of marital residence. This was also noted in anthropological investigations done between the 1950s and the 1970s [22–25,39]. Pumi is another ethnic group who speak Tibeto-Burman language in Yunnan and Sichuan Provinces, southwestern China. The total population is about 30 000. There are two branches of Pumi, based on the dialects: the southern Pumi mainly live in Lanping, Yunnan and northern Pumi live in Ninglang, Yunnan, the same area as Mosuo [39]. Pumi are normally patrilineal with either patrilocal or neolocal residence, but a small group of villages in Yongning area, Ninglang, Yunnan Province has adopted the matriliny and the duolocal residence norms of their Mosuo neighbours, while retaining their Pumi language [22–24,39].

Various researchers believed that the matriliny of Pumi in this area was due to the influence of the Mosuo population around them [22,24,39]. We also observed women of these matrilineal, duolocal Pumi married but not co-resident with Mosuo men, and vice versa, on a visit there in 2013. Wang and Yan studied the history of the Pumi village in Wenquan Town, Yongning, Yunnan Province and reported accounts of the switch between patriliny and matriliny that appeared about 100 years ago when the Mosuo aristocracy had relationships with Pumi women and began to encourage those Pumi to raise children outside of marriage (children who previously would have been at risk of infanticide under patrilineal kinship norms). Pumi people in Wenquan Town used to hold to a patrilineal genealogy, but most of those patrilineal genealogies were forgotten as duolocal residence and matriliny arose [24].

**Figure 1.**
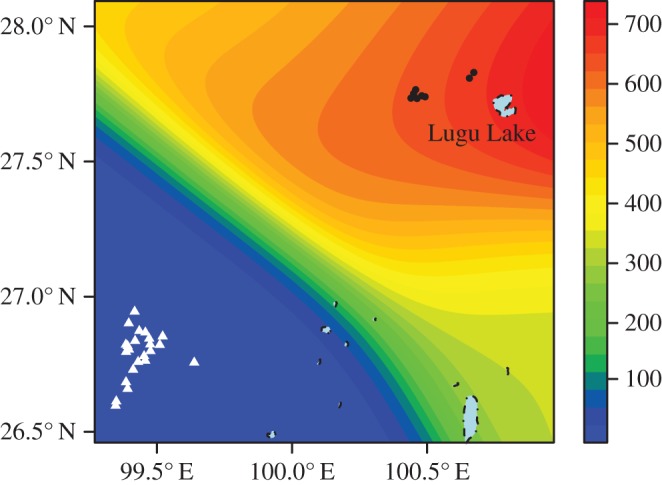
Historical locations of matrilineal Pumi and patrilineal Pumi populations and contour map of Mosuo populations around Lugu Lake between 1950s and 1970s. Black dots are predominantly matrilineal, duolocal Pumi villages and white triangles are predominantly patrilineal, patrilocal Pumi villages (following the normative kinship system of the Pumi in the region).

Historical data on population and kinship of Mosuo and Pumi in Sichuan and Yunnan can be found in published data, which were collected through the survey of ethnic minorities carried out in China between the 1950s and 1970s (electronic supplementary material, tables S1 and S2). Figure 1 shows a contour map of the Mosuo population in the area, where contours represent the numbers of Mosuo people in the area. The Pumi villages are marked with dots and triangles. It can be seen that the Pumi villages in the mainly Mosuo area are likely to contain duolocal households (black dots), whereas those Pumi villages in the mainly Pumi area (where there are no or very few Mosuo) (white triangles) do not.

Pumi males staying at home may not have found many females willing to disperse to live with them and ended up in ‘walking marriages’ with their children brought up in other households. Pumi females might find dispersal into a Mosuo household, already containing breeding females that are related to each other, very costly, so experienced higher pay-offs to staying in their natal household and having a duolocal marriage (Mosuo only rarely admit marriage partners into households, usually when they are experiencing a shortage of either males or females in the household). A large number of siblings not only increase the likelihood that someone will disperse but also reduce the chances that any marriage partner will move into a Mosuo household.

#### 3.1.2 Mosuo intermarriage with Han is reducing matrilineal, duolocality in favour of neolocality

**Figure 2.**
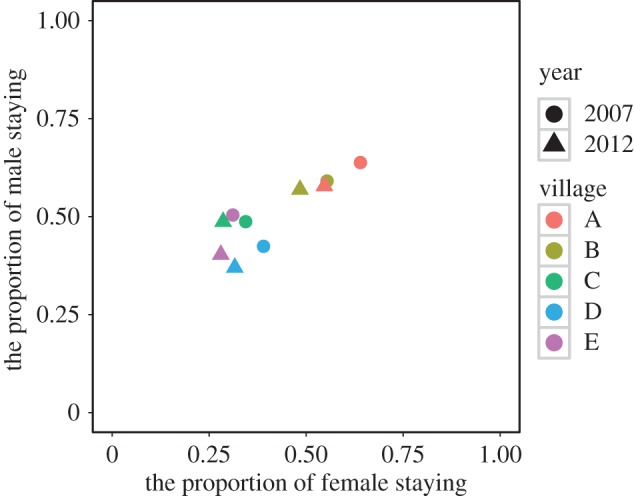
The proportion of females staying and males staying in their natal home after marriage in five villages (as shown in different colour) in Lugu Lake Town, Yanyuan County, Sichuan Province in 2007 (circles) and 2012 (triangles).

In 2007and 2012, we conducted demographic surveys in the area of Lugu Lake Town, Sichuan Province, China. Here patrilineal Han are now living among the Mosuo, both in small predominantly Han villages, and in mixed but predominantly Mosuo villages; and Mosuo and Han are intermarrying (electronic supplementary material, tables S3 and S4). Figure 2 suggests the area is on a trajectory that could be heading towards full neolocality.

The duolocal residence patterns in this area seem to have declined markedly between 2007 and 2012 (figure 2). About 30% of married Mosuo of both sexes had dispersed from their natal home in 2007, compared with over 40% in 2012. Among those that were intermarrying with Han those numbers increased to 60% in 2012 (electronic supplementary material, table S4). Intermarriage with Han was the most significant determinant of dispersal in married Mosuo females and males dispersing from their natal household (tables 1 and 2). Most Han of both sexes disperse at marriage with little change between 2007 and 2012 (electronic supplementary material, table S5). Intermarriage with Mosuo was actually associated with little change in the dispersal rate of the Han (electronic supplementary material, tables S6 and S7), but Han females marrying Mosuo males actually showed a small decline in dispersal (electronic supplementary material, table S6). It has been shown elsewhere on the Yunnan side of Lugu Lake that tourism is a major driver, as the economics of small nuclear households has been transformed by the arrival of the tourist industry [40]. It is interesting to note that in our main area of study (on the Sichuan side of the lake) over this time period, while tourism is also prevalent and increasing markedly, owning a tourist hotel was actually associated with Mosuo females and males being less likely to disperse (tables 1 and 2). Keeping cattle was also a predictor of Mosuo staying at home in both females and males. Increasing contact with and intermarriage with people from cultures with patrilocal or neolocal residence norms may be as or more important in this area than the economic changes brought about by tourism (tables 1 and 2). Increasing ease of travel may cause the kinship systems in a wider area to become more significant as individuals choose their mates from a geographically wider area. Duolocality is gradually being replaced by both sexes dispersing, generating neolocality.

**Table 1.** Logistic regression of determinants of having dispersed in married Mosuo females in 2012 as a function of age, years of education, household wealth, and intermarriage (*n*=1060). Significant effects are in italics. AIC=1368.7.

	estimate	s.e.	*z*-value	*Pr*(>|*z*|)	
(intercept)	−2.24203	0.751393	−2.984	0.002847	**
*age*	0.088928	0.02978	2.986	0.002825	**
*age square*	−0.00061	0.000288	−2.123	0.033724	*
years of education	0.027824	0.02684	1.037	0.299894	
*number of livestock*	−0.05717	0.010793	−5.297	1.18×10^−7^	***
*tourist income*	−0.4703	0.138051	−3.407	0.000658	***
intermarriage					
no intermarriage (ref)	0	0	0	0	
*intermarriage with Han*	0.839801	0.204389	4.109	3.98×10^−5^	***

Signif. codes: ‘***’ 0.001 ‘**’ 0.01 ‘*’ 0.05 ‘.’ 0.1 ‘ ’ 1.

**Table 2.** Logistic regression of determinants of having dispersed in married Mosuo males in 2012 as a function of age, years of education, household wealth and intermarriages (*n*=874). Significant effects are in italics. AIC=1066.

	estimate	s.e.	*z*-value	*Pr*(>|*z*|)	
(intercept)	−5.59575	1.157569	−4.834	1.34×10^−6^	***
*age*	0.188189	0.044339	4.244	2.19×10^−5^	***
*age square*	−0.00124	0.000412	−3.017	0.00255	**
year of education	0.004278	0.01929	0.222	0.82451	
*number of livestock*	−0.05453	0.011607	−4.698	2.62×10^−6^	***
*tourist income*	−0.44767	0.158801	−2.819	0.00482	**
intermarriage					
no intermarriage (ref)	0	0	0	0	
*intermarriage with Han*	1.168248	0.294204	3.971	7.16×10^−5^	***

Signif. codes: ‘***’ 0.001 ‘**’ 0.01 ‘*’ 0.05 ‘.’ 0.1 ‘ ’ 1.

### 3.2 Evolutionary game dynamics of post-marital residences

Based on the dynamical properties of asymmetric game [37,38], we can see that the time evolution of strategy S (D) in both females and males will mainly depend on the relative costs and benefits of each marital state (electronic supplementary material, Text). There are four possible cases with only one post-marital type that should be considered as evolutionarily stable (electronic supplementary material, figure S1*a*–*d*). More specifically, this will occur if one strategy always has higher pay-offs than the other one, irrespective of what the opposite sex chooses. For example, the strategy pair (FS, MS) (i.e. duolocality) is evolutionarily stable if females (males) who prefer to stay in their natal household after marriage have higher pay-offs than those who disperse, regardless of the decision by the other sex (electronic supplementary material, figure S1*a*). However, it is also possible that none of the post-marital types could be evolutionarily stable, in which the frequencies of strategies S and D in both female and male populations periodically change with an interior equilibrium called the centre in mathematics (electronic supplementary material, figure S2*a* and *b*). This situation could occur if for one sex the payoff is higher when both sexes follow the same strategy, but for the other sex the payoff is higher if both sexes follow different strategies.

**Figure 3.**
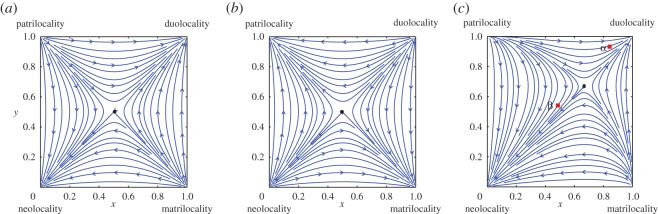
Time evolution of post-marital residence. The *x*-axis denotes the frequency of FS in female population, *y*-axis the frequency of MS in male population. Boundaries (0,0), (1,0), (0,1) and (1,1) represent the post-marital residences neolocality (FD, MD), matrilocality (FS, MD), patrilocality (FD, MS) and duolcality (FS, MS), respectively. The black dot denotes the unstable saddle point. (*a*) The boundaries (1,1) and (0,0) are locally asymmetrically stable, where the population within the basin of attraction of (0,0) will be attracted by the boundary neolocality, and the population within the basin of attraction of (1,1) will be attracted by the boundary duolocality. (*b*) The boundaries (1,0) and (0,1) are locally asymmetrically stable, where the population within the basin of attraction of (0,1) will be attracted by the boundary patrilocality, and the population within the basin of attraction of (1,0) will be attracted by the boundary matrilocality. (*c*) The boundaries (1,1) and (0,0) are locally asymmetrically stable, and neolocal residence has larger basin of attraction. The red square *α* represents the proportions of Mosuo females and males who stay in their natal household after marriage in 16 Mosuo villages around matrilineal Pumi villages in Yongning, Yunnan Province in 1950s (where *x*=0.82 and *y*=0.93; 660 females and 514 males; see electronic supplementary material, table S8 for the more detailed data). Similarly, the red square *β* represents the proportions of Mosuo females and males staying after marriage in five villages in Lugu Lake Town, Sichuan Province in 2007 (where *x*=0.49 and *y*=0.54; 911 females and 777 males). For model parameters, see the electronic supplementary material.

We are most interested in the situations where the evolution of post-marital residences not only depends on the pay-offs but also the system’s initial state (i.e. the initial frequencies of S and D females and males in the population). For these situations, the interior equilibrium of the system is an unstable saddle point. Both pairs where two sexes follow the same strategy (FS, MS) (duolocality) and (FD, MD) (neolocality) will be locally evolutionarily stable (figure 3*a*), or both pairs where two sexes follow a different strategy (FD, MS) (patrilocality) and (FS, MD) (matrilocality) will be locally evolutionarily stable (figure 3*b*). Hence if the best strategy for both female and male is to use the same strategy as the opposite sex, then the final state (or post-marital residence) will be attracted by either duolocality or neolocality. Which one will occur depends mainly on the initial state of the system. For example, a population within the basin of attraction of (0,0) in figure 3*a* will converge to neolocality (i.e. all couples will eventually live neolocally); while a population within the basin of attraction of (1,1) will converge to duolocality (i.e. there will ultimately only be duolocal marriages in the population). Similarly, if the best strategy for both female and male is to use the different strategy as the opposite sex, then the system final state will be either patrilocality or matrilocality (figure 3*b*).

The positions of the unstable saddle point in figure 3*a*,*b* are determined by the pay-offs. So it can move with the cost and benefit of each marital residence, and the basin of attraction changes (figure 3*c*). The scenario in figure 3*c* illustrates the situation that is likely to have occurred in the Pumi villages that are now duolocal (denoted by *α*), and that is probably occurring in the Mosuo population in Lugu Lake Town now (denoted by *β*).

Two features are thus made clear. Our model indicates that two post-marital types cannot coexist in the same population in the long run (assuming costs and benefits of each residence type are the same for each member of the population), with the initial local frequency of those staying and dispersing helping to determine the outcome. The minority type will generally be absorbed into the mainstream. Thus particular localities or interacting groups of people are likely to be characterized by a particular kinship system, hence generating the spatially and culturally discrete patterns of kinship norms that we tend to observe. Second, a change in the local frequency of male or female dispersers in a population could be enough to cause a norm to change, for example, from a matrilocal system to a patrilocal system, even if the costs and benefits of each of those marital residence systems remain unchanged. Thus, it may be that immigrants soon adopt the marital residence norms of those around them, or, if the immigrants are numerous enough, that they could cause the local norms to change. These frequency-dependent processes may thus either be a homogenizing force, maintaining the predominance of local marital residence norms even in the face of some immigration and intermarriage; or alternatively they can drive rarer local norms extinct in the face of extensive contact with a different culture.

## 4. Discussion and conclusion

While residence and marriage patterns do of course show some individual-level variation, broad cultural differences are clear. Indeed, the study of cultural differences in kinship was foundational in anthropology; and residence norms are one of those characteristics that tend to show persistent variation at the level of the cultural group. Frequency dependence is one mechanism by which group-level variation can be maintained, and here we show how this process might be occurring in kinship systems. We provide a model and real-world examples of how a kinship system in southwestern China may be switching between duolocality and neolocality in response to the frequency of the main dispersal strategy in the surrounding population.

The cost and benefit of disperse and stay may depend on the cooperation and competition between ego and co-residing kin. Humans are communal breeders. To maximize inclusive fitness, family members tend to help each other as well as compete with each other, if they share the same resource [14,17,41]. In this area, we have already shown that competition with kin and dispersal patterns can influence patterns of cooperation and competition [11,13,42]. Both sexes’ strategies of disperse and stay determine whose help they will receive and whom they are going to compete with.

Here we argue that non-dispersal by females, and thus the duolocal system with ‘walking marriage’ (where the men visit their partners at night but stay resident in their natal homes), arose when Pumi in these villages found themselves surrounded by a pool of potential Mosuo marriage partners that are not dispersers (given most Mosuo males and females expect to stay living in their natal houses).

Our models show that it is unlikely that any minority strategy can be maintained over the long term. Hence migration either into or out of an area can change kinship patterns in predictable ways. The rare duolocal system of the Mosuo may now only be sustainable in those few areas somewhat isolated from strong links with other Sino-Tibetan ethnic groups that are predominantly patrilocal or neolocal. However, both the movement of Han into the area and the increasing radius over which individuals find mates could be making the Mosuo duolocal kinship norms, where both sexes stay at home, become a minority kinship system, destined to eventually disappear in favour of neolocality. Cultural extinction is now accelerating all over the world, as groups become more connected; temporary migration becomes facilitated by roads and other forms of transport, communication over long distances becomes easier and more people move to urban centres in the face of economic development. Language is another frequency-dependent trait, and the number of languages spoken is showing a catastrophic decline in the face of these socio-economic changes, when compared with recent, historical linguistic diversity [43,44]. Kinship systems are not exactly analogous to language systems, as there is a strong functional component to family living arrangements whereas languages are likely to be neutral traits. However, a process of cultural homogenization, not dissimilar to that driving language mass extinction, is likely to be operating at the level of other types of cultural diversity, including a steady loss in the diversity of kinship systems, as a few prevalent norms become predominant to the exclusion of others. Thus, frequency-dependent selection on kinship traits may have been an evolutionary process that maintained cultural diversity in the face of some migration between groups, at a time when most of the population were relatively constrained in their geographical range. However, the same processes may now be acting as a homogenizing force depleting cultural diversity in the face of rapid socio-economic change.

## Supplementary Material

Supplementary Information for Ji et al.docx

## Supplementary Material

supplementary-other supporting data.xlsx, the first file contains model details and supporting data and other statistics, the second file contains the rest of supporting data
