# Supplementary material for: Kinship as a frequency dependent strategy
Source: R Soc Open Sci. 2016 Feb 17;3(2):150632. doi: 10.1098/rsos.150632 (PMC4785984; doi:10.1098/rsos.150632)
Supplement: Supplementary Information for Ji et al.docx [file rsos150632supp1.docx]

Supplementary Information for Ji et al Kinship as a frequency dependent strategy.

Kinship as a frequency dependent strategy.

Ting Ji^1,2^, Xiu-Deng Zheng^1^, Qiao-Qiao He^1^, Jia-Jia Wu^2^, Ruth Mace^2,*^ and Yi Tao^1,*^

^1^Key Laboratory of Animal Ecology and Conservation Biology, Centre for Computational and Evolutionary Biology, Institute of Zoology, Chinese Academy of Sciences, Beijing 100101, China

^2^Department of Anthropology, University College London, 14 Taviton St, London WC1H 0BW, UK

**^*^**Corresponding author: r.mace@ucl.ac.uk; yitao@ioz.ac.cn.

**Evolutionary game dynamics of post-marital residences (model details)**

The payoff matrix for females’ post-martial residence is given by $\mathbf{A}=\left(\begin{matrix} a_{SS} & a_{SD} \\ a_{DS} & a_{DD} \end{matrix}\right)$, where $a_{SS}$ ($a_{SD}$) denotes the payoff of a female displaying strategy FS when she married a male displaying strategy MS (MD), and $a_{DS}$ ($a_{DD}$) is the payoff of a female displaying FD when she married a male displaying strategy MS (MD). Similarly, for males, the payoff matrix for their post-marital residence is given by $\mathbf{B}=\left(\begin{matrix} b_{SS} & b_{SD} \\ b_{DS} & b_{DD} \end{matrix}\right)$, where $b_{SS}$ ($b_{SD}$) denotes the payoff of a male displaying strategy MS when he married with a female displaying strategy FS (FD), and $b_{DS}$ ($b_{DD}$) the payoff of a male displaying MD when he married with a female displaying strategy FS (FD). Through dynamics analysis of the bi-matrix game (i.e. asymmetric game) [36, 37], we investigate the time evolution of strategy S for both females and males.

If we assume that the population is large, marriage is monogamous, and that the chance a female meets a male is random, then the time evolution of frequency of strategy FS in females (denoted by $x$) and frequency of strategy MS in males (denoted by $y$) can be given by

$\begin{matrix} \frac{dx}{dt}=x\left(\left(\mathbf{Ay}\right)_{FS}-\mathbf{x}\cdot\mathbf{Ay}\right)=x(1-x)\left(\left(\mathbf{Ay}\right)_{FS}-\left(\mathbf{Ay}\right)_{FD}\right)\text{ }, \\ \frac{dy}{dt}=y\left(\left(\mathbf{Bx}\right)_{MS}-\mathbf{y}\cdot\mathbf{Bx}\right)=y(1-y)\left(\left(\mathbf{Bx}\right)_{MS}-\left(\mathbf{Bx}\right)_{MD}\right)\text{ }, \end{matrix}$ (1)

where $\mathbf{x}=(x,\text{ }1-x)$, $\mathbf{y}=(y,\text{ }1-y)$, $(\mathbf{Ay})_{FS}=ya_{SS}+(1-y)a_{SD}$ and $(\mathbf{Ay})_{FD}=ya_{DS}+(1-y)a_{DD}$ are the expected payoffs of strategies FS and FD, respectively, $(\mathbf{Bx})_{MS}=xb_{SS}+(1-x)b_{SD}$ and $(\mathbf{Bx})_{MD}=xb_{DS}+(1-x)b_{DD}$ are expected payoffs of strategies MS and MD, respectively, $\mathbf{x}\cdot\mathbf{Ay}=x(\mathbf{Ay})_{FS}+(1-x)(\mathbf{Ay})_{FD}$ is the average payoff of females, and $\mathbf{y}\cdot\mathbf{Bx}=y(\mathbf{Bx})_{MS}+(1-y)(\mathbf{Bx})_{MD}$ is the average payoff of males.

Notice that equation (1) is a standard dynamics for bi-matrix game (i.e. asymmetric game)[36, 37]. Thus, its main dynamical properties are:

1. One of the four boundaries is globally asymmetrical, no interior equilibrium exists.
   1. the boundary $(1,1)$ (duolocality) is globally asymmetrically stable if $a_{SS}>a_{DS}$, $a_{SD}>a_{DD}$, $b_{SS}>b_{DS}$ and $b_{SD}>b_{DD}$ (Supplementary Fig. 1a);
   2. the boundary $(0,0)$ (neolocality) is globally asymmetrically stable if $a_{SS}<a_{DS}$, $a_{SD}<a_{DD}$, $b_{SS}<b_{DS}$ and $b_{SD}<b_{DD}$ (Supplementary Fig. 1b);
   3. the boundary $(1,0)$ (matrilocality) is globally asymmetrically stable if $a_{SS}>a_{DS}$, $a_{SD}>a_{DD}$, $b_{SS}<b_{DS}$ and $b_{SD}<b_{DD}$ (Supplementary Fig. 1c),
   4. or the boundary $(0,1)$ (patrilocaity) is globally asymmetrically stable if $a_{SS}<a_{DS}$, $a_{SD}<a_{DD}$, $b_{SS}>b_{DS}$ and $b_{SD}>b_{DD}$ (Supplementary Fig. 1d);
2. if both $a_{SD}-a_{DD}$ and $a_{DS}-a_{SS}$ have same sign and both $b_{SD}-b_{DD}$ and $b_{DS}-b_{SS}$ have same sign, then there exists an interior equilibrium $\left(x*,y*\right)=\left(\frac{a_{SD}-a_{DD}}{a_{SD}-a_{DD}+a_{DS}-a_{SS}}\text{ },\text{ }\frac{b_{SD}-b_{DD}}{b_{SD}-b_{DD}+b_{DS}-b_{SS}}\right)$ (2)

with ;

- 1. the interior equilibrium point $\left(x*,\text{ }y*\right)$ is called a center if $\left(a_{SS}-a_{SD}-a_{DS}+a_{DD}\right)\left(b_{SS}-b_{SD}-b_{DS}+b_{DD}\right)<0$, and all orbits are periodic orbits surrounding $\left(x*,\text{ }y*\right)$ (Supplementary Fig. 2a and b); and
  2. the interior equilibrium point $\left(x*,\text{ }y*\right)$ is an unstable saddle point if $\left(a_{SS}-a_{SD}-a_{DS}+a_{DD}\right)\left(b_{SS}-b_{SD}-b_{DS}+b_{DD}\right)>0$, where both boundaries $(1,1)$ and $(0,0)$ are locally asymmetrically stable if $\left(a_{SS}-a_{SD}-a_{DS}+a_{DD}\right)$ and $\left(b_{SS}-b_{SD}-b_{DS}+b_{DD}\right)$ are positive (Fig. 3a), or both boundaries $(1,0)$ and $(0,1)$ are locally asymmetrically stable if $\left(a_{SS}-a_{SD}-a_{DS}+a_{DD}\right)$ and $\left(b_{SS}-b_{SD}-b_{DS}+b_{DD}\right)$ are negative (Fig. 3b) (The parameters for Figure 3 Panel a are taken as $a_{SD}-a_{DD}=-1$, $a_{DS}-a_{SS}=-1$, $b_{DS}-b_{SS}=-1$, $b_{SD}-b_{DD}=-1$; for Panel b are taken as $a_{SD}-a_{DD}=1$, $a_{DS}-a_{SS}=1$, $b_{DS}-b_{SS}=1$, $b_{SD}-b_{DD}=1$; and for Panel c are $a_{SD}-a_{DD}=-1$, $a_{DS}-a_{SS}=-0.5$, $b_{DS}-b_{SS}=-1$ and $b_{SD}-b_{DD}=-0.5$.For the more detailed mathematical analysis, see Hofbauer and Sigmund, 1998.).

The dynamical properties of equation (1) imply that each of four possible types of post-marital residence could be an ESS (evolutionarily stable strategy) pair, denoted by $(\hat{x},\hat{y})$ (for $\hat{x}=0,1$ and $\hat{y}=0,1$), if $\mathbf{x}\cdot\mathbf{A}\left(\hat{x},\text{ }1-\hat{x}\right)^{T}<\left(\hat{x},\text{ }1-\hat{x}\right)\cdot\mathbf{A}\left(\hat{x},\text{ }1-\hat{x}\right)^{T}$ and $\mathbf{y}\cdot\mathbf{B}\left(\hat{y},\text{ }1-\hat{y}\right)^{T}<\left(\hat{y},\text{ }1-\hat{y}\right)\cdot\mathbf{B}\left(\hat{y},\text{ }1-\hat{y}\right)^{T}$ for all possible $x\neq\hat{x}$ and $y\neq\hat{y}$. Thus, it is easy to see that the strategy pair (FS, MS) (i.e. duolocality) is an ESS if $a_{SS}-a_{DS}>0$ and $b_{SS}-b_{DS}>0$, (FS, MD) (i.e. matrilocality ) is an ESS if $a_{SS}-a_{DS}>0$ and $b_{DD}-b_{SD}>0$, (FD, MS) (i.e. patrilocaity) is an ESS if $a_{DD}-a_{SD}>0$ and $b_{SS}-b_{DS}>0$, and (FD, MD) (i.e. neolocality) is an ESS if $a_{DD}-a_{SD}>0$ and $b_{DD}-b_{SD}>0$. On the other hand, it is also easy to see that the interior equilibrium point $\left(x*,\text{ }y*\right)$ cannot be an ESS if it exists[36].

Particularly, if the interior equilibrium point $\left(x*,\text{ }y*\right)$ exists, then the dynamics of equation (1) will strongly depend on its initial state. For example, if $\left(x*,\text{ }y*\right)$ is a unstable saddle point and both $\left(a_{SS}-a_{SD}-a_{DS}+a_{DD}\right)$ and $\left(b_{SS}-b_{SD}-b_{DS}+b_{DD}\right)$ are positive, then system state will be attracted by the boundary $(1,1)$ ($(0,0)$) if the initial state is near $(1,1)$ ($(0,0)$) (Fig. 3a); and, similarly, if $\left(x*,\text{ }y*\right)$ exists and both $\left(a_{SS}-a_{SD}-a_{DS}+a_{DD}\right)$ and $\left(b_{SS}-b_{SD}-b_{DS}+b_{DD}\right)$ are negative, then the system state will be attracted by the boundary $(1,0)$ ($(0,1)$) if the initial state is near $(1,0)$ ($(0,1)$) (Fig. 3b).

More specifically, when males prefer to stay in their natal household after marriage, females who also choose to stay in their natal household and have marriage duolocally will have higher payoffs than those who disperse and live with husband’s family (), while if males prefer to disperse after marriage, females who also disperse and live neolocally will have higher payoffs than those who choose to stay in their natal household with their husbands moving in (). Meanwhile, duolocal males have a higher payoff than patrilocal males (), and neolocal males have a higher payoff than matrilocal males (). Under this condition, the best strategy for both female and male is following what the opposite sex chooses. Then all orbits converge to neolocality or duolocality (Fig. 3a). If patrilocal females have a higher payoff than duolocal females, matrilocal females have a higher payoff than neolocal females, matrilocal males have a higher payoff than duolocal males, and patrilocal males have a higher payoff than neolocal males due to change of benefit and cost of post-marital residence, i.e. $a_{DS}>a_{SS},a_{SD}>a_{DD}$ and $b_{\text{DS}}>b_{SS},b_{\text{SD}}>b_{DD}$, then the best strategy for both females and males is to use a different strategy from the opposite sex. All orbits converge to matrilocality or patrilocality (Fig. 3b).

Thus, our model predicts that the post-marital residence in a population can be frequency dependent.

**Supplementary Figure 1 and legend**

Supplementary Figure 1 Evolution of post-marital residence. X is the proportion of females adopting the FS (stay) strategy, and y is the proportion of males adopting the MS (stay) strategy in the population. (0,0), (1,0), (0,1) and (1,1) represent the four boundary equilibrium points of post-marital residence neolocality (FD, MD), matrilocality (FS, MD), patrilocality (FD, MS) and duolcality (FS, MS). A shows duolocality is globally asymmetrically stable; b shows neolocality is globally asymmetrically stable; c shows matriloclaity is globally asymmetrically stable; and d shows patrilocality is globally asymmetrically stable.

**Supplementary Figure 2 and legend**

Supplementary Figure 2 Evolution of post-marital residence. X is the proportion of females adopting the S (stay) strategy, and y is the proportion of males adopting the S strategy in the population. (0,0), (1,0), (0,1) and (1,1) represent of the four boundary equilibrium points of post-marital residence neolocality (DD), matrilocality (SD), patrilocality (DS) and duolcality (SS). Black points show the unstable saddle point. A and b show that the frequencies of S and D change either clockwise or counter-clockwise in the population.

Supplementary Table 1 Historical matrilineal and patrilineal Pumi villages in Yunnan Provinces. Data are form published data collected through the survey of ethnic minorities carried out in China between the 1950s and 1970s. Populations are listed as numbers.

| Village | Number of households | Matriliny /duolocal present | Reference |
| --- | --- | --- | --- |
| 1 | 40 | No | 22 |
| 2 | 30 | No | 22 |
| 3 | 18 | No | 22 |
| 4 | 10 | No | 22 |
| 5 | 30 | No | 22 |
| 6 | Na | No | 22 |
| 7 | 40 | No | 22 |
| 8 | 17 | No | 22 |
| 9 | 24 | No | 22 |
| 10 | 44 | No | 22 |
| 11 | 38 | No | 22 |
| 12 | 23 | No | 22 |
| 13 | 8 | No | 22 |
| 14 | 5 | No | 22 |
| 15 | 42 | No | 22 |
| 16 | 12 | No | 22 |
| 17 | 17 | No | 22 |
| 18 | 31 | No | 22 |
| 19 | 10 | No | 22 |
| 20 | 20 | No | 22 |
| 21 | 15 | No | 22 |
| 22 | 10 | No | 22 |
| 23 | 20 | No | 22 |
| 24 | 20 | Yes | 21 |
| 25 | 13 | Yes | 24 |
| 26 | 15 | Yes | 24 |
| 27 | 15 | Yes | 21 |
| 28 | 15 | Yes | 21 |
| 29 | 11 | Yes | 21 |
| 30 | 9 | Yes | 21 |
| 31 | 44 | Yes | 23 |
| 32 | 10 | Yes | 21 |

Supplementary Table 2 Historical Mosuo population in Sichuan and Yunnan Provinces. Data are form published data collected through the survey of ethnic minorities carried out in China between the 1950s and 1970s. Villages are listed as numbers within the general area.

| County | Population | Mosuo population | Reference |
| --- | --- | --- | --- |
| Yanyuan | 1 | 2717 | 36 |
|  | 2 | 283 | 36 |
| Muli | 3 | 492 | 36 |
| Ninglang | 4 | 1057 | 40 |
|  | 5 | 1088 | 33 |
|  | 6 | 349 | 39 |
|  | 7 | 181 | 43 |
|  | 8 | 318 | 37 |
|  | 9 | 338 | 38 |
|  | 10 | 546 | 24 |
|  | 11 | 756 | 34 |
|  | 12 | 228 | 35 |
|  | 13 | 226 | 41 |
|  | 14 | 1632 | 42 |
|  | 15 | 215 | 21 |
|  | 16 | 79 | 23 |
| Lijiang | 17 | 116 | 42 |
| Lanping | 18 | 0 | 22 |
|  | 19 | 0 | 22 |
|  | 20 | 0 | 22 |
|  | 21 | 0 | 22 |
|  | 22 | 0 | 22 |
|  | 23 | 0 | 22 |
|  | 24 | 0 | 22 |
|  | 25 | 0 | 22 |

Supplementary Table 3 Population of Mosuo and Han in Lugu Lake Town. Data are from Table of population information of all villages in Lugu Lake Town, collected by office of family planning of Lugu Lake Town government in 2012.

| Ethnic group  Village | Mosuo | Han | Han/All |
| --- | --- | --- | --- |
| A | 1107 | 753 | 0.4048 |
| B | 1795 | 138 | 0.0714 |
| C | 1055 | 73 | 0.0647 |
| D | 775 | 537 | 0.4093 |
| E | 896 | 453 | 0.3358 |

Supplementary Table 4 Number of staying at natal household or dispersing to a different household after marriage in Mosuo people in Lugu Lake town.

| Sex | Type | 2007 | | 2012 | | | | | |
| --- | --- | --- | --- | --- | --- | --- | --- | --- | --- |
|  |  | S | D | D% | S | D | D% |  | |
| Female | Not intermarried | 551 | 307 | 0.3578 | 514 | 384 | 0.4276 | |  |
|  | Intermarried with Han | 119 | 64 | 0.3497 | 75 | 111 | 0.5968 | |  |
| Male | Not intermarried | 515 | 260 | 0.3355 | 459 | 351 | 0.4333 | |  |
|  | Intermarried with Han | 55 | 29 | 0.3452 | 38 | 52 | 0.5778 | |  |

S: stay at natal household after marriage; D: disperse from the natal household after marriage.

Both intermarried females and males dispersed more during the five years than non-intermarried females.

Supplementary Table 5 Number of staying at natal household or dispersing to a differentfing to a different both females and males. household after marriage in Han people in Lugu Lake town.

| Sex | Type | 2007 | | | | 2012 | | | |
| --- | --- | --- | --- | --- | --- | --- | --- | --- | --- |
|  |  | S |  | D | D% | S |  | D | D% |
| Female | Not intermarried | 35 |  | 208 | 0.8560 | 11 |  | 235 | 0.9553 |
|  | Intermarried with Mosuo | 17 |  | 34 | 0.6667 | 4 |  | 49 | 0.9245 |
| Male | Not intermarried | 69 |  | 126 | 0.6462 | 73 |  | 125 | 0.6313 |
|  | Intermarried with Mosuo | 19 |  | 36 | 0.6545 | 17 |  | 40 | 0.7018 |

S: stay at natal household after marriage; D: disperse from the natal household after marriage.

Supplementary Table 6 Logistic regression analysis: determinants of having dispersed by 2012 for married Han females as a function of age, year of education, household wealth, and intermarriage with Mosuo (n=208). Significant effects are in bold. AIC: 110.67.

|  | Estimate | Std. Error | z value | Pr(>|z|) |
| --- | --- | --- | --- | --- |
| (Intercept) | -2.193738 | 3.171466 | -0.692 | 0.489 |
| Age | 0.205952 | 0.137698 | 1.496 | 0.135 |
| Agesq | -0.001852 | 0.001384 | -1.338 | 0.181 |
| Edu | 0.153081 | 0.10921 | 1.402 | 0.161 |
| Livestock | -0.021396 | 0.023474 | -0.911 | 0.362 |
| Income | -0.640964 | 0.601619 | -1.065 | 0.287 |
| Intermarriage |  |  |  |  |
| no intermarriage (ref) | 0 | 0 | 0 | 0 |
| intermarriage with Mosuo | -0.646449 | 0.667329 | -0.969 | 0.333 |

Signif. codes: 0 ‘***’ 0.001 ‘**’ 0.01 ‘*’ 0.05 ‘.’ 0.1 ‘ ’ 1

Supplementary Table 7 Logistic regression analysis: determinants of having dispersed by 2012 for married Han males as a function of age, year of education, household wealth, and intermarriage with Mosuo (n=204). Significant effects are in bold. AIC: 247.93.

|  | Estimate | Std. Error | z value | Pr(>|z|) |
| --- | --- | --- | --- | --- |
|  | Estimate | Std. Error | z value | Pr(>|z|) |
| (Intercept) | -3.1771003 | 2.2064503 | -1.44 | 0.1499 |
| Age | 0.1021595 | 0.0934935 | 1.093 | 0.2745 |
| Agesq | -0.0003662 | 0.0009679 | -0.378 | 0.7052 |
| Edu | 0.0139575 | 0.0443377 | 0.315 | 0.7529 |
| Livestock | -0.0433899 | 0.0335045 | -1.295 | 0.1953 |
| Income | 0.2823294 | 0.4009626 | 0.704 | 0.4814 |
| Intermarriage |  |  |  |  |
| no intermarriage (ref) | 0 | 0 | 0 | 0 |
| Intermarriage with Mosuo | 0.7276687 | 0.4046041 | 1.798 | 0.0721 |

Signif. codes: 0 ‘***’ 0.001 ‘**’ 0.01 ‘*’ 0.05 ‘.’ 0.1 ‘ ’ 1

Supplementary Table 8 number of staying and dispersing males and females after marriage in 16 Mosuo villages around matrilineal pumi villages in Yongning District, Yunnan.

| village no. | female | | male | | references |
| --- | --- | --- | --- | --- | --- |
|  | stay | disperse | stay | disperse |  |
| 1 | 63 | 12 | 45 | 5 | 40 |
| 2 | 46 | 6 | 31 | 3 | 40 |
| 3 | 41 | 7 | 25 | 2 | 40 |
| 4 | 21 | 2 | 15 | 3 | 40 |
| 5 | 21 | 6 | 20 | 1 | 40 |
| 6 | 18 | 3 | 21 | 1 | 40 |
| 7 | 24 | 7 | 24 | 3 | 33 |
| 8 | 13 | 2 | 11 | 0 | 33 |
| 9 | 24 | 3 | 26 | 0 | 33 |
| 10 | 16 | 7 | 15 | 0 | 33 |
| 11 | 17 | 7 | 18 | 2 | 33 |
| 12 | 63 | 11 | 39 | 6 | 33 |
| 13 | 37 | 16 | 37 | 4 | 34 |
| 14 | 34 | 7 | 27 | 2 | 34 |
| 15 | 47 | 7 | 58 | 0 | 34 |
| 16 | 59 | 13 | 66 | 4 | 35 |
| total | 544 | 116 | 478 | 36 |  |
